# Supplementary material for: Cerebrospinal Fluid Chloride Is Associated with Disease Activity of Relapsing–Remitting Multiple Sclerosis: A Retrospective Cohort Study
Source: Brain Sci. 2023 Jun 7;13(6):924. doi: 10.3390/brainsci13060924 (PMC10296568; doi:10.3390/brainsci13060924)
Supplement: Supplementary file 1 [file brainsci-13-00924-s001.zip › brainsci-2412115-SI.pdf]

Table S1: Correlation between CSF findings and the Multiple Sclerosis Severity Score at the time of the first lumbar puncture stratifying patients by maintenance therapies

We examined the relationship between CSF findings and Multiple Sclerosis Severity Score at the time of the first lumbar puncture stratifying by maintenance therapies. we found a positive correlation between CSF WBC and MSSS in the not-DMT group (r_s = 0.28; p = 0.049).

Variable	Non-DMT		DMT	
	Correlation coefficient	*p*-value	Correlation coefficient	*p*-value
CSF chloride	0.01	0.925	0.27	0.170
CSF glucose	-0.04	0.803	0.16	0.424
CSF WBC	0.28	0.049	0.12	0.533
CSF protein	0.07	0.611	-0.04	0.841
Serum chloride	0.02	0.865	-0.14	0.481
Serum glucose	0.03	0.838	0.15	0.432
Serum protein	0.08	0.587	-0.07	0.733
Serum WBC	-0.09	0.552	0.08	0.671
CSF/serum chloride	0.03	0.820	-0.30	0.120
CSF/serum glucose	0.02	0.906	-0.10	0.614
CSF/serum protein	-0.08	0.574	0.03	0.875

WBC, white blood cell; CSF, cerebrospinal fluid; DMT, Disease-modifying therapies.

Table S2: Correlation between CSF findings and the Multiple Sclerosis Severity Score at the first relapse stratifying patients by maintenance therapies

We examined the relationship between CSF findings and Multiple Sclerosis Severity Score at the first relapse stratifying by maintenance therapies. we found a positive correlation between CSF WBC and MSSS in the not-DMT group ($r_s = 0.31$; $p = 0.030$).

Variable	Non-DMT		DMT	
	Correlation coefficient	*p*-value	Correlation coefficient	*p*-value
CSF chloride	-0.03	0.834	0.13	0.518
CSF glucose	0.19	0.201	0.16	0.412
CSF WBC	0.31	0.030	-0.17	0.374
CSF protein	0.10	0.497	-0.03	0.889
Serum chloride	0.06	0.690	-0.24	0.221
Serum glucose	-0.09	0.529	0.10	0.612
Serum protein	0.15	0.313	-0.06	0.757
Serum WBC	-0.13	0.364	0.14	0.475
CSF/serum chloride	0.08	0.605	-0.20	0.304
CSF/serum glucose	-0.21	0.140	-0.18	0.366
CSF/serum protein	-0.11	0.445	0.05	0.788

WBC, white blood cell; CSF, cerebrospinal fluid; DMT, Disease-modifying therapies.

Table S3: CSF findings baseline feature in patients with the presence or absence of OCB

We examined the relationship between CSF findings and the presence of CSF OCBs at presentation. There was no significant difference between the CSF findings of patients with positive OCBs and those without positive OCBs.

Variable	OCB-, n = 39a	OCB+, n = 38 [a]	p Value [b]
CSF chloride	123.1 ± 2.5	123.4 ± 2.6	0.614
CSF glucose	3.4 (3.1, 3.7)	3.3 (3.1, 3.9)	0.935
CSF protein	0.2 (0.2, 0.3)	0.2 (0.2, 0.3)	0.959
CSF WBC	2.0 (0.0, 7.0)	2.0 (0.0, 6.0)	0.696
Serum glucose	4.6 (4.2, 6.0)	4.9 (4.5, 5.3)	0.586
Serum chloride	103.7 ± 3.2	104.4 ± 2.0	0.258
Serum protein	64.4 (60.2, 67.8)	64.8 (61.4, 66.8)	0.907
Serum WBC	8.0 (6.9, 10.7)	6.4 (5.0, 7.7)	0.003
CSF/serum chloride	0.8 ± 0.0	0.8 ± 0.0	0.533
CSF/serum glucose	1.4 (1.2, 1.7)	1.5 (1.3, 1.7)	0.956
CSF/serum protein	289.3 (191.5, 396.0)	285.7 (220.9, 348.4)	0.996

[a] Median (IQR); Mean ± SD

[b] Wilcoxon rank sum test; Welch two sample t-test

Abbreviations: CSF, cerebrospinal fluid; OCB, oligoclonal bands; WBC, white blood cells.

Table S4: Univariate and multivariate Cox regression analyses for recurrent events stratifying patients by the presence or absence of OCB

We examined the relationship between CSF findings and the presence of CSF OCBs at presentation. An elevated CSF chloride, CSF WBC count, and CSF glucose were associated with a high risk of relapse in the multivariate stratifying patients by the presence or absence of OCB.

Variable	Univariate		Multivariate [a]	
	HR (95% CI)	*p* Value	HR (95% CI)	*p* Value
CSF chloride	1.16(1.06-1.27)	0.001	1.13(1.05-1.22)	0.001
CSF glucose	1.45(1.15-1.82)	0.002	1.45(1.22-1.73)	<0.001
CSF protein	0.29(0.02-5.43)	0.407	0.95(0.06-14.87)	0.973
CSF WBC	1.04(0.99-1.10)	0.108	1.06(1.02-1.10)	0.005
Serum glucose	1.05(0.88-1.25)	0.601	1.01(0.88-1.15)	0.917
Serum chloride	1.02(0.89-1.16)	0.784	1.01(0.93-1.10)	0.828
Serum protein	1.05(1.01-1.09)	0.021	1.04(1.01-1.07)	0.005
Serum WBC	0.99(0.90-1.08)	0.776	0.97(0.90-1.05)	0.501
CSF/serum chloride	0.01(0.01-271.02)	0.249	0.01 (0.01-10.03)	0.138
CSF/serum glucose	0.70(0.34-1.44)	0.330	0.59(0.35-1.02)	0.057
CSF/serum protein	1.00(1.00-1.00)	0.007	1.00(1.00-1.00)	0.052

[a] The multivariate model was adjusted for sex and age at disease onset, maintenance therapies.

Abbreviations: CSF, cerebrospinal fluid; OCB, oligoclonal bands; WBC, white blood cells; CI, confidence interval; HR, hazards ratio.
